# Showcasing the Saudi e-referral system experience: the epidemiology and pattern of referrals utilising nationwide secondary data

**DOI:** 10.3389/fmed.2024.1348442

**Published:** 2024-06-27

**Authors:** Nawfal A. Aljerian, Abdullah A. Alharbi, Reem S. AlOmar, Meshary S. Binhotan, Hani A. Alghamdi, Mohammed S. Arafat, Abdulrahman Aldhabib, Mohammed K. Alabdulaali

**Affiliations:** ^1^Medical Referrals Centre, Ministry of Health, Riyadh, Saudi Arabia; ^2^Emergency Medicine Department, King Saud bin Abdulaziz University for Health Sciences, Riyadh, Saudi Arabia; ^3^Family and Community Medicine Department, Faculty of Medicine, Jazan University, Jazan, Saudi Arabia; ^4^Department of Family and Community Medicine, College of Medicine, Imam Abdulrahman Bin Faisal University, Dammam, Saudi Arabia; ^5^Emergency Medical Services Department, College of Applied Medical Sciences, King Saud bin Abdulaziz University for Health Sciences, Riyadh, Saudi Arabia; ^6^King Abdullah International Medical Research Centre, Riyadh, Saudi Arabia; ^7^Department of Family and Community Medicine, College of Medicine, King Saud University, Riyadh, Saudi Arabia; ^8^Ministry of Health, Riyadh, Saudi Arabia

**Keywords:** epidemiology, e-referral, health policy, e-health systems, public health

## Abstract

**Introduction:**

Referrals are an integral part of any healthcare system. In the Kingdom of Saudi Arabia (KSA) an electronic referral (e-referral) system known as the Saudi Medical Appointments and Referrals Centre (SMARC) began formally functioning in 2019. This study aims to showcase the Saudi experience of the e-referral system and explore the epidemiology of referrals nationally.

**Methods:**

This retrospective descriptive study utilised secondary collected data between 2020 and 2021 from the SMARC system. Cross tabulations with significance testing and colour-coded maps were used to highlight the patterns across all regions.

**Results:**

The study analysed over 600,000 referral requests. The mean age of patients was 40.70 ± 24.66 years. Males had a higher number of referrals (55.43%). Referrals in 2021 were higher than those in 2020 (56.21%). Both the Autumn and Winter seasons had the highest number of referrals (27.09% and 27.43%, respectively). The Surgical specialty followed by Medicine had the highest referrals (26.07% and 22.27%, respectively). Life-saving referrals in the Central region were more than double those in other regions (14.56%). Emergency referrals were also highest in the Southern regions (44.06%). The Central and Eastern regions had higher referrals due to unavailable sub-speciality (68.86% and 67.93%, respectively). The Southern regions had higher referrals due to both unavailable machine and unavailable beds (18.44% and 6.24%, respectively).

**Conclusion:**

This study shows a unique system in which referrals are between secondary, tertiary, and specialised care. It also highlights areas of improvement for equitable resource allocation and specialised care in slightly problematic areas as well as the use of population density in future planning.

## Introduction

Digital health is transforming healthcare into real-time, individualised care, enhancing diagnosis, treatment, and patient empowerment (1). It provides opportunities beyond conventional healthcare for prevention, early illness detection, and chronic disease management (2). However, literature shows mixed results of this digital transformation across different countries (3). In new medicine, digital technologies can reinforce best practices like electronic referrals (4).

E-referrals are critical for providing quality healthcare. Efficient referral systems promote collaboration across all levels of care (5). Referral system success depends on many factors including patient barriers, resources, technology, and patient behaviour (6).

Saudi Arabia has recently undergone significant healthcare reforms and system changes as part of the National Transformation Programme launched in 2015 under Saudi Vision 2030. This aims to provide equitable, high-quality healthcare for all through innovations such as a robust digital health infrastructure (7–9). One key component of the digital health transformation is the establishment of a national electronic referral system known as the Saudi Medical Appointments and Referrals Centre (SMARC). SMARC facilitates referrals between healthcare facilities across all levels of care in the Kingdom. It utilises a Unified System of Medical Referrals (USMR) to receive and coordinate referral requests nationally through a centralised platform (10).

Whilst Saudi Arabia has made significant progress in implementing digital health, few studies have evaluated the impacts and effectiveness of these efforts. One study found preparedness amongst Saudi facilities for adapting to Vision 2030 changes was varied (11). Understanding patterns and utilisation of the new e-referral system across regions can provide insights into its performance and areas needing improvement.

The e-referral system within the Kingdom of Saudi Arabia (KSA), previously known as Ehalati, faced challenges when initially launched in 2012 including fragmented systems across hospitals, lack of integration between public and private facilities, and inadequate expertise in digital health solutions. The information technology platform at that time lacked features like artificial intelligence, robust data analytics, and interoperability, making centralised data management difficult. With many hospitals relying on their own individual platforms, doctors often depended on informal referral networks to coordinate care. However, aligned with Vision 2030, the centralised electronic referral system was revamped and fully reimplemented in 2019 as the SMARC (12).

Since 2019, substantial improvements have been made with Ministry of Health (MoH) support to transition to a unified, national e-referral platform. Targeted training programmes were implemented to build digital health capabilities across facilities. The SMARC system leverages advanced health information technologies like artificial intelligence and predictive analytics for improved care coordination. By standardising the e-referral platform and workflows across all public and private hospitals, SMARC addressed fragmentation and seamlessly integrates referrals digitally. With all governmental and majority of private healthcare facilities now connected to the centralised system, SMARC facilitates efficient nation-wide referral management and represents a major milestone in the digital transformation of Saudi Arabia’s health sector.

Other countries have also implemented effective digital health systems, such as Catalonia’s electronic health information exchange which has been a European leader since 2009. This system enabled critical health data sharing during the COVID-19 pandemic (13). Additionally, the European Health Data Space (EHDS) promotes individuals’ electronic health data access and use for research and public benefit (14). Global digital health initiatives like Catalonia’s and the EHDS exemplify how digital systems can improve health outcomes and research. Lessons from these efforts can inform Saudi Arabia’s digital health advancements under Vision 2030.

Saudi Arabia currently serves a population of almost 34 million through a combination of public and private facilities across 13 administrative regions. As part of Vision 2030 reforms, the healthcare system is being upgraded to boost quality, efficiency and value through integrating public and private sectors. This includes establishing five new business units to manage the 13 healthcare regions alongside national insurance companies, overseen by the MoH and new insurance centres (15–17).

This study is the first to showcase the KSA’s nationwide referral patterns using routine data from the new SMARC e-referral system. Examining referral epidemiology and trends will provide insights into the system’s effectiveness and inform future optimisations to enhance its impact as a key digital health initiative under Vision 2030.

## Materials and methods

### Setting and data source

Under the new healthcare transformation adopted by the MoH, the 13 administrative areas will be pooled into five BUs as follows; Asir, Jazan, and Najran in the Southern BU; Aljouf, Hail, Northern Border and Tabuk in the Northern BU; Riyadh and Alqassim in the Central BU; Makkah, Medina, and Albaha in the Western BU, and the Eastern administrative area in the Eastern BU (15).

All hospitals have a designated coordination department usually known as the Office of Coordination and Eligibility for Treatment (OCET), which has access to the USMR. The OCET receives a referral request from the treating physician which is then uploaded to the USMR. Depending on a patient’s medical condition, the referral request is uploaded as either lifesaving, emergency, or routine. These three types of referrals are categorised by SMARC to facilitate the referral process, and to timely secure acceptance to patients who are in most need.

For emergency and routine referrals, the OCET has the privilege to choose up to three hospitals that can potentially offer the needed service at the same region, or alternatively the USMR will automatically choose three appropriate hospitals. The SMARC system has built-in timeframes for referral requests to be accepted, depending on the urgency. For emergency referrals, hospitals have 72 h to accept the request, whilst routine referrals have a 14-day timeframe. If the initially chosen hospitals reject the request within the allotted timeframe, the request is sent to additional hospitals for consideration. If no hospital has accepted the referral request once the timeframe elapses, the case is escalated to the SMARC medical referral management team. They will find an appropriate alternative from the pool of public and private hospitals, searching both within the same region and in other regions if needed. Importantly, whilst awaiting referral acceptance, patients continue receiving necessary healthcare management at the sending hospital to ensure stability until the transfer is arranged.

To expedite the referral request for life-threatening cases, SMARC offers a 24-h lifesaving hotline (1937) in which any treating physician can call directly. The call is answered by a SMARC lifesaving agent and directed to an on-call medical consultant for review and acceptance. If the request is accepted as a lifesaving by the on-call consultant, the treating physician through the OCET, will upload the request to the USMR along with the acceptance code and the name of the receiving hospital. Treating physicians who requested emergency referrals can also call and use this service when patients’ health conditions deteriorate whilst waiting the emergency referral acceptance.

**Figure 1:**
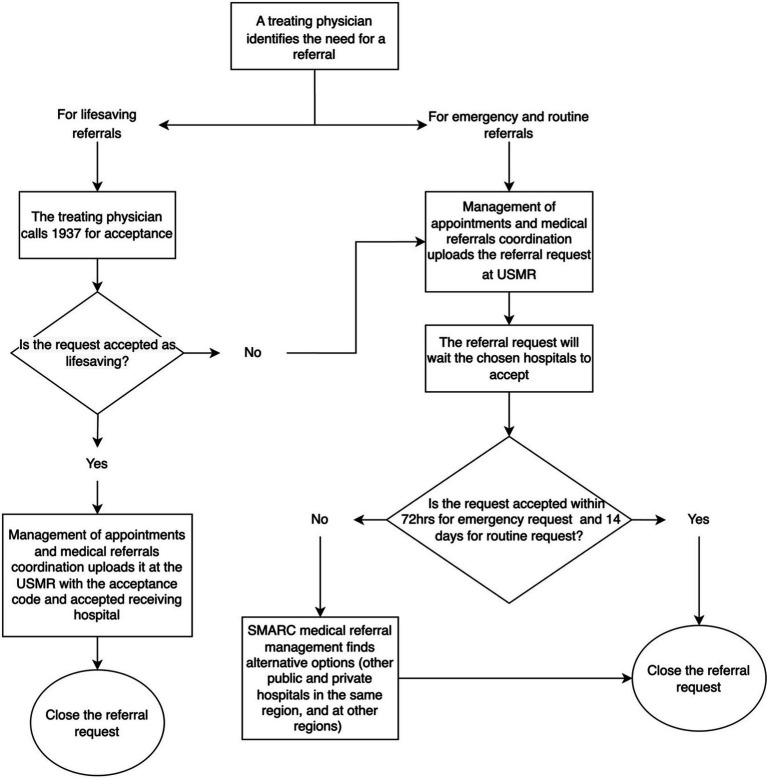
The process of referral acceptance across Saudi regions.

Additionally, SMARC oversees referrals for Saudi patients seeking to return to the KSA for treatment. For these cases, Saudi Embassies abroad have access to the USMR and may initiate a referral request. Figure 1 describes the process of the referral requests acceptance.

This study utilised routinely collected secondary data extracted from the SMARC e-referral system database between 2020 and 2021. Permission was obtained to access and analyse this de-identified dataset for research purposes, which was provided by the SMARC team after obtaining necessary approvals. The informed consent was waived given the retrospective nature of this study which relied solely on anonymized secondary data. The dataset was checked for completeness and consistency, and any incomplete or inconsistent records were removed prior to analysis. No personally identifiable information was included to maintain patient confidentiality. The variables included in the dataset are described in the Measurements section.

### Study design

This retrospective study utilised secondary routinely collected data provided by the SMARC e-referral system. The dataset includes all referral requests submitted through the SMARC system nationally in 2020 and 2021, with no exclusion criteria applied.

### Ethical considerations

Both the MoH and Imam Abdulrahman Bin Faisal University institutional review boards have approved this study (23-77-E) and (IRB-2023-01-305). Standard precautions were taken to protect the confidentiality and privacy of patients’ data involved.

### Measurements

The dataset includes variables on sex, age, date of referral (month and year), type of referral (e.g., lifesaving, routine), bed type (e.g., ward bed or burn bed), reason for referral (e.g., unavailable speciality or unavailability of a specialised physician), medical speciality requesting the referral (e.g., medicine or surgery), region of referral request according to the five business units of the New Model of Care as well as according to the entire 13 administrative regions of the country.

### Statistical analysis

To answer the objectives of the study, cross tabulations of explanatory variables according to the five BUs were performed, and tests of significance through Chi-squared tests and ANOVA tests were computed where appropriate. All analyses were run using the Stata statistical software version 16 (18). To further study the distribution of referral requests across the 13 administrative areas, colour-coded maps were drawn in ArcGIS (GIS software) version 10.0 (19), according to the percentage of referrals of each area.

## Results

### Sociodemographic characteristics of patients

Table 1 presents the sociodemographic characteristics of all patients. The total number of patients was 671,672 with an average age of 40.70 ± 24.66 years. Over 55% of referrals were for males. Non-Saudi’s made up 15.11% of the total referrals. Most referral requests originated from the Western BU (34.99%), and the least originated from the Eastern BU (11.02%). Referrals were higher in 2021 compared to 2020 (56.21% and 43.79%, respectively).

**Table 1:** Sociodemographic characteristics of patients with referral requests.

Characteristics	Total (%) 671,672 (100.00)
Age (μ, SD)	36.88 (23.40)
**Gender**	
Males	372,308 (55.43)
Females	299,364 (44.57)
**Nationality**	
Non-Saudi	101,474 (15.11)
Saudi	570,198 (84.89)
**Region (BUs)**	
Central	101,793 (15.16)
Eastern	74,018 (11.02)
Western	235,020 (34.99)
Northern	118,212 (17.60)
Southern	142,629 (21.23)
**Year**	
2020	294,114 (43.79)
2021	377,558 (56.21)

### Pattern of referrals across months

**Figure 2:**
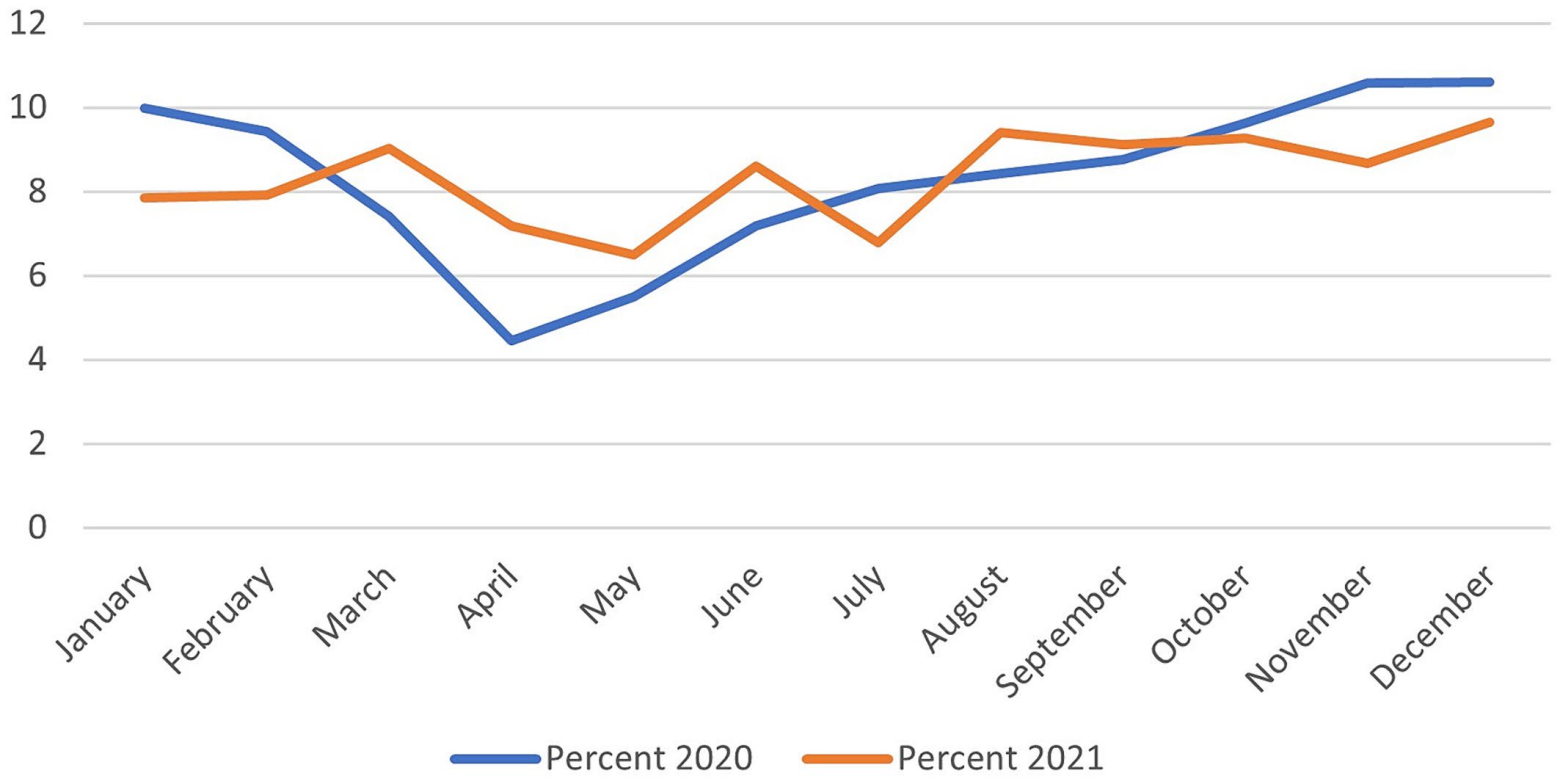
Pattern of monthly e-referrals for the years 2020 and 2021 across the Kingdom of Saudi Arabia.

Upon examining the overall monthly pattern of referrals in Figure 2, both years of 2020 and 2021 have commenced with a high percentage of referrals, dipping to their lowest levels in April 2020 and May 2021. In 2020, the chart displays an initial decline in medical case referrals between February and March. From the beginning of April onwards, there is a significant graduate increase in referrals through to the year end. In contrast, 2021 displays a less consistent trend with more fluctuations and a significant increase in referrals in March, June, August, and December. Comparatively, referral rates from both years meet by the end of the year, indicating an expected new standard for medical referrals has developed.

### Patterns across medical specialties

**Figure 3:**
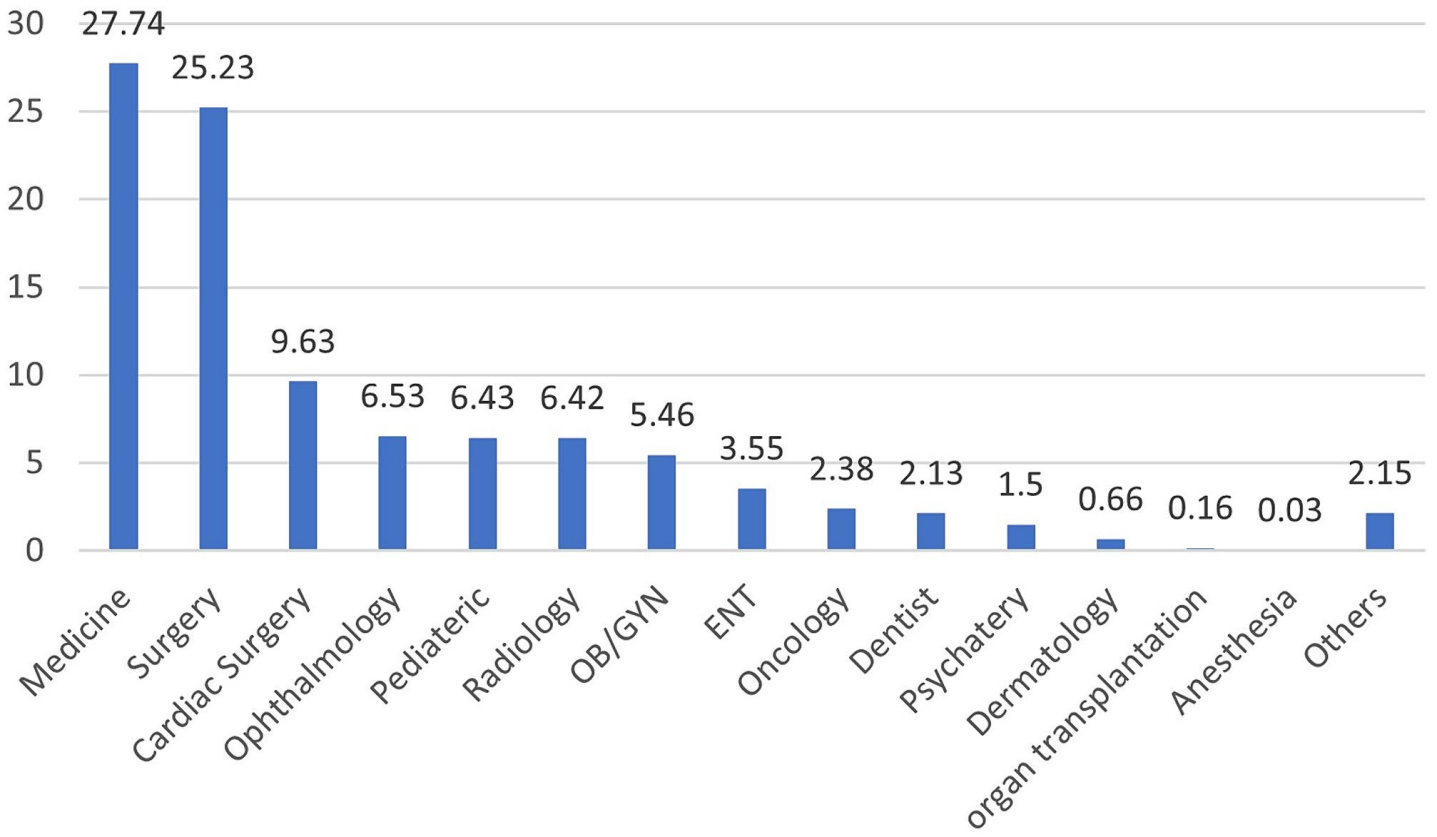
The e-referral requests by medical speciality for 2020 and 2021 across the Kingdom of Saudi Arabia.

Figure 3 shows the pattern of medical specialties requesting referrals. Patients with referrals pertaining to internal medicine were the most common, reaching over a quarter of all requests (27.74%). Followed by general surgery and cardiac surgery (25.23% and 9.63%). The least common referral requests were for anaesthesia (0.03%).

### Sociodemographic variables and region of referral request

Associations between sociodemographic variables and the region of referral request are presented in Table 2. Referrals originating from the Western and Southern regions were for patients who were relatively older than those from other regions (average age 42.38 and 41.63 years). Males dominated referrals from the Southern regions (59.18%), whereas for females, referrals were similarly high for both the Central and the Eastern regions. Requests for non-Saudis was highest in the Western region and lowest in the Eastern region (19.46% and 9.06%, respectively). All associations were significant at the 0.05 level.

**Table 2:** Associations between sociodemographic variables and region of referral request.

Characteristics	Central 101,793 (15.16)	Eastern 74,018 (11.02)	Western 235,020 (34.99)	Northern 118,212 (17.60)	Southern 142,629 (21.23)
Age (μ, SD)	36.38 (22.46)	36.58 (23.06)	38.79 (23.48)	34.75 (23.55)	36.00 (23.77)
*P-*value	<0.001				
**Gender**					
Males	52,851 (51.92)	38,423 (51.91)	134,196(57.10)	62,546 (52.91)	84,408 (59.18)
Females	48,942 (48.08)	35,595 (48.09)	100,824 (42.90)	55,666(47.09)	58,221(40.82)
*P-*value	<0.001				
**Nationality**					
Non-Saudi	12,734 (12.51)	6,707(9.06)	45,737(19.46)	12,182 (10.31)	24,114 (16.91)
Saudi	89,059 (87.49)	67,311(90.94)	189,283 (80.54)	106,030 (89.69)	118,515(83.09)
*P-*value	<0.001				

### Referral characteristics and region of referral request

The Central region had the highest number of referrals due to life saving events (14.56%), whereas the Northern region had the lowest at only 2.32%. For routine outpatient referrals, those originating from the Northern region were the highest reaching 57.39%. Emergency related referrals were most common in the Southern region and least common in the Eastern region (44.06% and 25.94%, respectively). As for dialysis, no referrals were registered from the Eastern region, whereas the Western region had 93 requests.

Also, 44.85% of referrals for ward beds were found to be in the Southern region. The Western region had the highest requests for isolation beds (5.63%). Requests for ICU beds was highest in the Central region (9.36%), whereas for CCU beds it was highest in the Western region (3.26%). As for PICU and NICU beds, they were highest in the Northern and Western regions, respectively.

As for reasons for referral, unavailable subspeciality was the most common reason and was highest in the Central region followed by the Eastern region (68.86% and 67.93%, respectively). The unavailability of a specialised physician was mostly reported in the Northern region (24.03%). The Southern region mostly reported the unavailability of a machine and the unavailability of a bed compared to all other regions (18.44% and 6.24%, respectively). Referrals due to social reasons were most commonly reported in the Western region, whilst there were 213 referrals due to a royal order from the Eastern region. Referrals due to injuries were only reported in the Southern region, whereas referrals due to health crises were highly reported in the Western region. All associations were significant at the 0.05 level (Table 3).

**Table 3:** The e-referral characteristics and region of referral requests in 2020 and 2021 across the Kingdom of Saudi Arabia.

Characteristic	Total 671,672 (100.00)	Central 101,793 (15.16)	Eastern 74,018 (11.02)	Western 235,020 (34.99)	Northern 118,212 (17.60)	Southern 142,629 (21.23)
**Referral types**						
Life saving	47,315 (7.04)	14,820 (14.56)	3,793 (5.12)	16,516 (7.03)	2,748 (2.32)	9,438 (6.62)
Routine OPD	317,484 (47.27)	51,944)51.03(	42,333 (57.19)	100,378 (42.71)	67,846 (57.39)	54,983 (38.55)
Routine inpatient	85,955 (12.80)	7,997 (7.86)	8,693 (11.74)	38,150 (16.23)	15,760 (13.33)	15,355 (10.77)
ER	220,802 (32.87)	27,030 (26.55)	19,199 (25.94)	79,883 (33.99)	31,854 (26.95)	62,836 (44.06)
Dialysis	116 (0.02)	2 (0.00)	0 (0.00)	93 (0.04)	4 (0.00)	17 (0.01)
*P-*value		<0.001				
**Bed type**						
OPD no bed	316,152 (47.07)	51,691 (50.78)	42,209 (57.03)	99,938 (42.52)	67,654 (57.23)	54,660 (38.32)
Ward	242,731 (36.14)	33,239 (32.65)	22,552 (30.47)	87,203 (37.10)	35,762 (30.25)	63,975 (44.85)
Burning bed	630 (0.09)	97 (0.10)	65 (0.09)	254 (0.11)	71 (0.06)	143 (0.10)
Isolation bed	27,067 (04.03)	1,742 (1.71)	2,557 (3.45)	13,225 (5.63)	3,024 (2.56)	6,519 (4.57)
ICU	47,217 (07.03)	9,528 (9.36)	4,247 (5.74)	19,505 (8.30)	4,825 (4.08)	9,112 (6.39)
CCU	18,603 (02.77)	2,360 (2.32)	1,094 (1.48)	7,653 (3.26)	3,067 (2.59)	4,429 (3.11)
PICU	8,102 (01.21)	1,392 (1.37)	659 (0.89)	2,702 (1.15)	1,664 (1.41)	1,685 (1.18)
NICU	11,170 (01.66)	1,744 (1.71)	635 (0.86)	4,540 (1.93)	2,145 (1.81)	2,106 (1.48)
*P-*value		<0.001				
**Reason of referral**						
Unavailable subspecialty	413,619 (61.38)	70,300 (68.86)	50,429 (67.93)	144,614 (61.33)	68,582(57.82)	79,694 (55.68)
Unavailable physician	114,882 (17.05)	17,736 (17.35)	13,233 (17.78)	35,724 (15.17)	28,482 (24.03)	19,706 (13.76)
Unavailable machine	89,790 (13.33)	10,932 (10.74)	5,938 (7.95)	29,420 (12.49)	17,080 (14.40)	26,361 (18.44)
Unavailable bed	24,305 (03.61)	1,817 (01.77)	1,956 (02.62)	10,057 (04.27)	1,537 (01.29)	8,921 (06.24)
Social reason	1,662 (0.25)	95 (0.09)	328 (0.44)	1,111 (0.47)	100 (0.08)	28 (0.02)
Health crisis	27,414 (04.06)	1,209 (01.17)	2,233 (02.99)	14,678 (06.24)	2,785 (02.34)	6,509 (04.55)
*P-*value		<0.001				

### Total referral requests and referrals received by administrative areas

**Figure 4:**
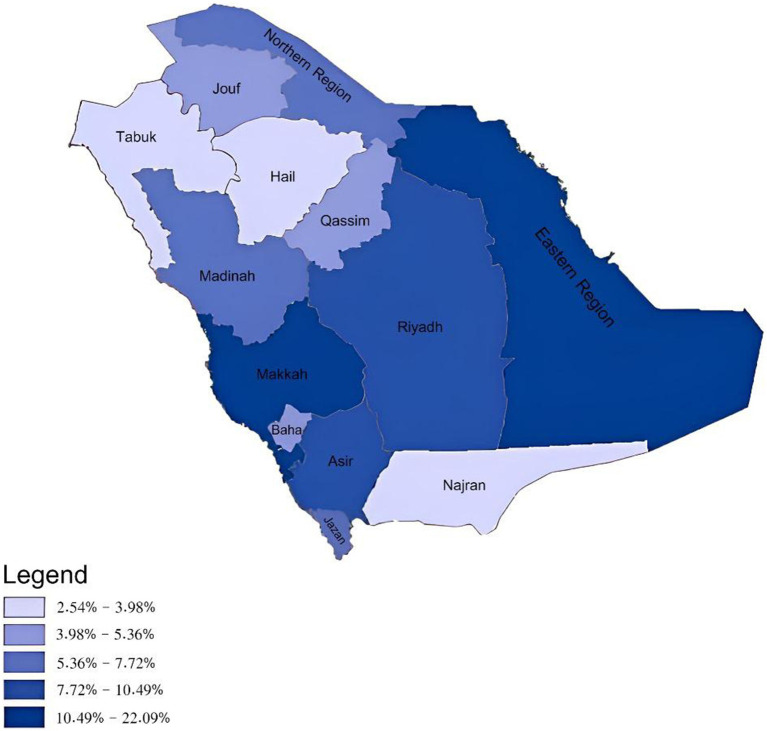
Total e-referral requests sent according to the 13 administrative areas of the Kingdom of Saudi Arabia.

Both the Eastern and Makkah administrative areas were in the highest quintile with requests beyond 10.49% for both. However, Hail, Tabuk, and Najran administrative areas were within the lowest quintiles (Figure 4).

**Figure 5:**
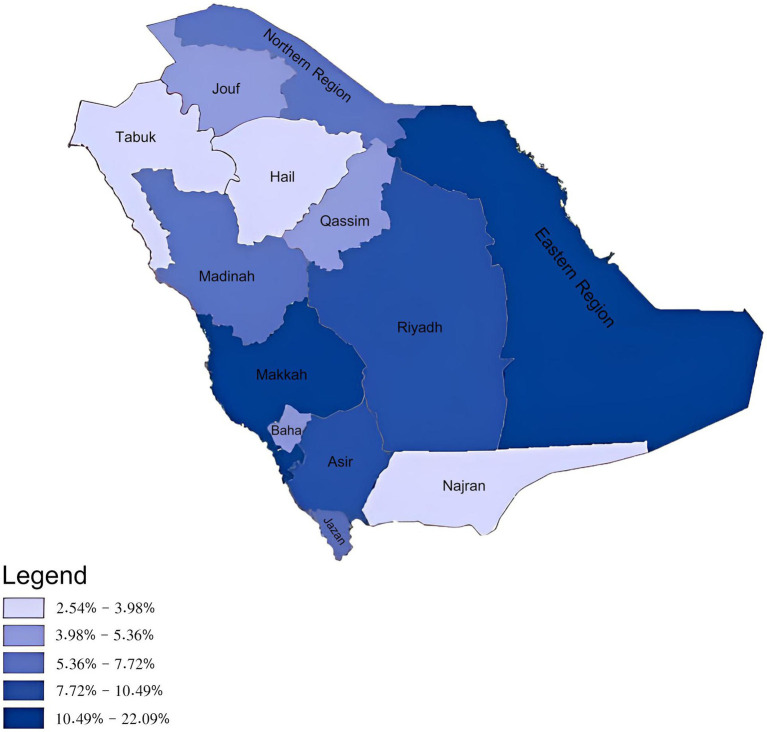
Total e-referral requests received according to the 13 administrative areas of the Kingdom of Saudi Arabia.

As for receiver areas, both Riyadh and Makkah were within the highest quintile both reaching above 12.80% of the total requests received. Whereas, Hail, Najran and the Northern areas were amongst the lowest (Figure 5).

## Discussion

This study is the first to present the current status of the Saudi e-referral system. It also explored the patterns of e-referrals across the country utilising routinely collected data stored by the SMARC system. Patterns of referrals have been enormously studied worldwide (20–23). However, making clear comparisons are likely to be difficult due to differences between countries in, for example, local contexts and health care systems (24). Also, patterns of referrals in the current literature were mostly limited to primary healthcare referrals (20–23). This contrasts with the SMARC system in the KSA, which is concerned with secondary, tertiary, and specialised levels of care only. Also, this analysis of the Saudi e-referral system provides the first empirical evidence of inequalities across the different BUs. Previous studies in the KSA have shown that there are discrepancies in the quality of treatment provided to COVID-19 patients amongst the five different BUs at the outbreak’s onset (15). Other several noteworthy observations can be drawn from the findings of this study.

Sex variations in e-referrals suggest the presence of disparities in healthcare-seeking patterns. Higher referrals amongst males are reflected in the higher proportion of males compared to females as shown in the 2022 census (25). However, sex variations were observed in referrals in other countries including America and Canada (23, 26, 27).

The observed discrepancy in the ratio of Saudi/non-Saudi patients may be ascribed to the inherent characteristics of the healthcare system and expatriates’ situation in the KSA. Expatriates are primarily in the country for work purposes and are obligated to be medically fit in order to have a work visa. Also, since employers are required to provide health insurances for their foreign employees, most of them attain health services from private hospitals. This is despite the fact that free healthcare is provided to all citizens regardless of nationality in MoH facilities especially during the COVID-19 pandemic (28).

Regional variations in referral patterns likely stem from differences in healthcare resources and infrastructure. The uneven distribution of healthcare services across and within regions is well-established (29–33). Disparities between the five new business units in Saudi Arabia have been noted in prior studies on quality indicators for COVID-19 patients (15, 34, 35). Our analysis provides further evidence of disproportionality amongst the BUs regarding referral initiation and receipt.

The Eastern region and Makkah initiated the highest total number of referral requests. Contributing factors could include their greater population density (25) and regional health system capacities.

However, when examining referral request rates per 10,000 population, the Northern and Albaha regions were actually highest (Supplementary Table 1). This suggests medical resource limitations, also reflected when grouped into their respective Northern and Western BUs. Conversely, the Eastern region had the second lowest rate *per capita* despite having the most total referrals, highlighting the need to consider population density in resource allocation.

Riyadh and Makkah had the most referral requests. As the country’s major healthcare hubs with advanced facilities and specialties (36), these regions likely attract more referrals due to advanced medical capabilities. Similar regional differences have been observed elsewhere globally (26). Further research into the distribution of health system resources, such as workforce and facilities, is needed to fully explain the variations in medical referrals across Saudi Arabia’s regions.

Internal medicine emerged as the most commonly referring speciality highlighting the prevalence of chronic diseases and cardiovascular conditions in the population (37). Surgical related specialities followed which may be due to shortage of surgical staff, particularly in surgical sub-specialities. Patients and referring physicians often prefer and trust specialised centres, further driving the demand for surgical services (38). Comparatively, in Canada, dermatology was one of the top referred specialties, whereas in the KSA, dermatology related referrals were low (39).

One notable finding is the high number of referrals due to unavailable subspecialties, which is particularly high in the Central and Eastern regions. This may be an indication of a shortage of certain sub-specialities, where despite the fact that these areas are home to two of the main cities of the country namely Riyadh and Dammam, which both include excellent healthcare services and high quality of care, these cities are surrounded by smaller towns with hospitals equipped with lesser specialised staff referring to those main cities. It may also indicate that healthcare staff within those areas pursue a high-quality of care (40). Also, the Northern region stands out with a significant proportion of referrals attributed to the unavailability of a specialised physician, indicating a potential need for improved access to specialised care in that region. In contrast, the Southern region reports a higher frequency of referrals due to the unavailability of a machine and bed, indicating infrastructure-related challenges. Additionally, it is worth noting that referrals due to injuries were exclusively reported in the Southern region. This suggests that the Southern region may have a higher prevalence of injury-related incidents or a greater need for specialised care for injuries compared to other regions. The Western region shows a higher occurrence of referrals due to social reasons, potentially reflecting the influence of social and cultural factors on healthcare-seeking behaviour.

Discrepancies in bed types across regions indicates potential differences in healthcare needs and allocation of resources. The Southern region exhibits a relatively higher number of referrals for ward beds, this may be attributed to the concentration of general hospitals or specialised facilities within that particular geographic location. The assignment of distinct bed categorisations, such as burn beds and isolation beds, may be a result of various factors, such as the prevalence of illnesses in a specific geographical region, the demand for specialised medical treatments, and the demographic attributes of the populace.

Lastly, referral rates are influenced by national and international incidents. In 2020, COVID-19 pandemic and its consequences including the lockdown could explain the low referral rate in 2020 compared to 2021. The drop in referrals during the pandemic has also been seen in different settings such as emergency departments, and heart diseases in countries including Italy and the United Kingdom (41, 42). However, this is the first study to observe the influence on a national level. With the implementation of the new regional healthcare transformations under the 2030 Vision and the merging of the existing 13 regions into five BUs; this research promises to encourage greater dedication to increasing the outstanding quality and equitable distribution of healthcare services.

Current findings show a momentary view of national referral patterns during a two-year time frame. These results provide an opportunity for improvement in terms of equity in resource allocation as well as enhancement of specialised care especially in problematic areas highlighted here. Furthermore, the use of this nationwide secondary data enabled us to explore the patterns of e-referrals across the country. However, certain limitations should be addressed. The reliance on secondary data obtained from the e-referral system limits the scope of variables examined. Also, the absence of similar studies in the wider literature makes direct comparisons challenging. Additionally, the study’s focus on referral patterns may overlook other important aspects of healthcare, such as primary care utilisation or patient outcomes. Future research should address these limitations to provide a more comprehensive understanding of healthcare utilisation and effectiveness.

Additionally, several key insights for healthcare systems worldwide can be drawn from the evolution of SMARC. First, a unified e-health platform not only enhances service quality and efficiency but also improves access, conserves resources and eliminates service redundancies. Second, centralised tracking allows effective monitoring of health outcomes and resource utilisation, which aids in the identification of strengths and weaknesses within the system. Finally, this integrated approach increases strategic resource distribution, informs health policy and advances academic research, leading to greater optimization of healthcare delivery (43, 44). Investments in e-health and digital health provide economic benefits as well through streamlining operations and reducing administrative burdens, both of which are achieved by automating processes and minimising the need for in-person consultations. Digital health technologies improve diagnostic and treatment accuracy, improving patient outcomes and reducing medical errors; and extend service reach, particularly in underserved areas, maximising resource utilisation. These benefits contribute to an overall improvement in the efficiency of the healthcare system, ultimately leading to lower costs over the long-term (43, 44).

## Conclusion

This study examined the underlying mechanism of an important telehealth tool, namely, e-referrals. Certain patterns were observed which included higher referrals for males, as well as in internal medicine and surgical related specialities, and unavailable subspeciality being the most commonly reported reason for referrals. We recommend the use of population density in the future planning of resource allocation and specialised care.

## Data availability statement

The data analysed in this study is subject to the following licenses/restrictions: the data was requested from the Ministry of Health. Any researcher can also request this data provided they have an ethical approval. Requests to access these datasets should be directed to https://www.moh.gov.sa.

## Ethics statement

Ethical approval for the study was obtained from the Ministry of Health’s Institutional Review Board (23-77-E) and Imam Abdulrahman Bin Faisal’s Institutional Review Board (IRB-2023-01-305). The informed consent was waived given the retrospective nature of this study which relied solely on anonymized secondary data.

## Author contributions

NA: Conceptualization, Data curation, Supervision, Writing – review & editing. AAlh: Conceptualization, Data curation, Formal analysis, Investigation, Methodology, Project administration, Supervision, Validation, Visualization, Writing – original draft, Writing – review & editing. RA: Formal analysis, Investigation, Methodology, Validation, Visualization, Writing – original draft, Writing – review & editing. MB: Investigation, Validation, Visualization, Writing – original draft, Writing – review & editing. HA: Project administration, Validation, Visualization, Writing – original draft, Writing – review & editing. MAr: Conceptualization, Validation, Visualization, Writing – review & editing. AAld: Conceptualization, Validation, Visualization, Writing – review & editing. MAl: Conceptualization, Project administration, Validation, Visualization, Writing – review & editing.
