# Supplementary material for: Showcasing the Saudi e-referral system experience: the epidemiology and pattern of referrals utilising nationwide secondary data
Source: Front Med (Lausanne). 2024 Jun 27;11:1348442. doi: 10.3389/fmed.2024.1348442 (PMC11238632; doi:10.3389/fmed.2024.1348442)
Supplement: Supplementary file 1 [file Table_1.docx]

**Supplementary - Table 1: Referral Request Rates per 10,000 Population Across Business Units and Administrative Regions in KSA**

| **Business Units** | | **Number of requests** | **Total population (%)**  **32,175,224 (100.00)** | **Rate per 10,000** |
| --- | --- | --- | --- | --- |
| **Central BU** | | 101,793 (15.16) | 9,927,927 (30.85) | 102.53 |
| **Western BU** | | 235,020 (34.99) | 10,498,620 (32.62) | 223.85 |
| **Eastern BU** | | 74,018 (11.02) | 5,125,254 (15.93) | 144.41 |
| **Southern BU** | | 142,629 (21.23) | 4,021,582 (12.50) | 354.65 |
| **Northern BU** | | 118,212 (17.60) | 2,601,841 (8.01) | 454.33 |
| **Business Units** | **Administrative area** | **Number of requests** | **Total population**  **(%)**  **3,217,5224**  **(100.00)** | **Rate per 10,000** |
| **Central** | **Riyadh** | 704,59 (10.49) | 8,591,748 (26.70) | 82.00 |
|  | **AL Qassim** | 313,34 (4.67) | 1,336,179 (4.15) | 234.50 |
| **Western** | **Makkah** | 148,397 (22.09) | 8,021,463 (24.93) | 184.99 |
|  | **Madinah** | 518,53 (7.72) | 2,137,983 (6.64) | 242.53 |
|  | **Albaha** | 347,70 (5.18) | 339,174(1.54) | 1,025.13 |
| **Eastern** | **Eastern region** | 740,18 (11.02) | 5,125,254 (15.92) | 144.41 |
| **Southern** | **Aseer** | 703,28 (10.47) | 2,024,285 (6.29) | 347.42 |
|  | **Jazan** | 492,42 (7.33) | 1,404,997 (4.36) | 350.47 |
|  | **Najran** | 230,59 (3.43) | 592,300 (1.84) | 389.31 |
| **Northern** | **Aljouf** | 360,09 (5.36) | 595,822 (1.85) | 604.35 |
|  | **Northern Border** | 384,24 (5.72) | 373,577 (1.16) | 1,028.54 |
|  | **Tabuk** | 267,14 (3.98) | 886,036 (2.75) | 301.50 |
|  | **Hail** | 170,65 (2.54) | 746,406 (2.31) | 228.62 |
